# Gypenosides Alleviate Cone Cell Death in a Zebrafish Model of Retinitis Pigmentosa

**DOI:** 10.3390/antiox10071050

**Published:** 2021-06-29

**Authors:** Xing Li, Reem Hasaballah Alhasani, Yanqun Cao, Xinzhi Zhou, Zhiming He, Zhihong Zeng, Niall Strang, Xinhua Shu

**Affiliations:** 1 School of Basic Medical Sciences, Shaoyang University, Shaoyang 422000, China; lixing1114@163.com (X.L.); cyq057@163.com (Y.C.); 40003@hnsyu.edu.cn (Z.H.); 2 Department of Biological and Biomedical Sciences, Glasgow Caledonian University, Glasgow G4 0BA, UK; rhhasani@uqu.edu.sa (R.H.A.); Xinzhi.Zhou@gcu.ac.uk (X.Z.); 3 Department of Biology, Faculty of Applied Science, Umm Al-Qura University, Makkah 21961, Saudi Arabia; 4 College of Biological and Environmental Engineering, Changsha University, Changsha 410022, China; z20181201@ccsu.edu.cn; 5 Department of Vision Science, Glasgow Caledonian University, Glasgow G4 0BA, UK; N.Strang@gcu.ac.uk

**Keywords:** retinitis pigmentosa, gypenosides, cone cell death, rpgrip1, zebrafish, network pharmacology

## Abstract

Retinitis pigmentosa (RP) is a group of visual disorders caused by mutations in over 70 genes. RP is characterized by initial degeneration of rod cells and late cone cell death, regardless of genetic abnormality. Rod cells are the main consumers of oxygen in the retina, and after the death of rod cells, the cone cells have to endure high levels of oxygen, which in turn leads to oxidative damage and cone degeneration. Gypenosides (Gyp) are major dammarane-type saponins of *Gynostemma pentaphyllum* that are known to reduce oxidative stress and inflammation. In this project we assessed the protective effect of Gyp against cone cell death in the *rpgrip1* mutant zebrafish, which recapitulate the classical pathological features found in RP patients. Rpgrip1 mutant zebrafish were treated with Gyp (50 µg/g body weight) from two-months post fertilization (mpf) until 6 mpf. Gyp treatment resulted in a significant decrease in cone cell death compared to that of untreated mutant zebrafish. A markedly low level of reactive oxygen species and increased expression of antioxidant genes were detected in Gyp-incubated mutant zebrafish eyes compared to that of untreated mutant zebrafish. Similarly, the activities of catalase and superoxide dismutase and the level of glutathione were significantly increased in Gyp-treated mutant zebrafish eyes compared to that of untreated mutant zebrafish. Gyp treatment also decreased endoplasmic reticulum stress in *rpgrip1* mutant eyes. Expression of proinflammatory cytokines was also significantly decreased in Gyp-treated mutant zebrafish eyes compared to that of untreated mutant zebrafish. Network pharmacology analysis demonstrated that the promotion of cone cell survival by Gyp is possibly mediated by multiple hub genes and associated signalling pathways. These data suggest treatment with Gyp will benefit RP patients.

## 1. Introduction

Retinitis pigmentosa (RP) is a group of inherited retinal disorders, affecting approximately 1 in 4000 adults worldwide [1]. Most RP cases, including the autosomal dominant, autosomal recessive and X-linked forms, are non-syndromic with dysfunction only occurring in the retina. However, some RP cases, such as Usher syndrome and Bardet-Biedl syndrome, are syndromic with defects occurring in other tissues [2]. Mutations in over 70 genes have been reported to cause RP (RetNet; https://sph.uth.edu/retnet/, accessed on 20 April 2021). The retinitis pigmentosa GTPase regulator (RPGR) gene is the most common X-linked RP-causing gene. The RPGR protein directly or indirectly interacts with other ciliary proteins to form a protein complex, which promotes ciliary protein trafficking in the photoreceptors [3]. One of the RPGR interacting partners, the RPGR interacting protein 1 (RPGRIP1) gene is also known to mutate in patients with RP [4], cone-rod dystrophy [5] or Leber congenital amaurosis (LCA) [6,7]. Loss of RPGRIP1 protein causes defects in ciliogenesis in the human retinal pigment epithelial (RPE) cell line (RPE1) and both abnormal development of the rod outer segments and early retinal degeneration in mice and zebrafish [8,9,10].

RP is characterized by initial rod cell death and late cone cell degeneration. RP patients experience night blindness at an early stage; with tunnel vision developing as a result of rod cell death. Central vision loss develops at a later stage as a result of cone cell degeneration. The products of RP-associated genes demonstrate diverse functions, which involve the visual cycle, phototransduction, maintenance of photoreceptors and regulation of transcription. As a result, the death of rod cells is linked to distinct processes associated with light damage, endoplasmic reticulum stress, metabolic stress, ciliary protein trafficking defect and abnormal mRNA processing [11]. Cone cells start to degenerate when almost all rod cells are dead. This degeneration is thought to result from cone cells being subjected to an increasing production of reactive oxygen species (ROS), due to the high levels of oxygen being delivered from choroidal capillary circulation. This results from the choroidal vessels being unable to autoregulate the blood supply when photoreceptor oxygen consumption is decreased due to the death of the rod cells [2]. High levels of oxidative damage have also been reported in the cone cells of the transgenic RP pig model following rod degeneration [12]. These findings suggest that cone cell degeneration could be reduced by limiting oxidative damage. Several studies have also shown that administration of antioxidants preserve cone function and slow cone death in RP mouse models (Rd1, Rd10 and RHO^Q344ter^ transgenic line) [13,14,15]. NRF2 is the master regulator that controls expression of antioxidant proteins and triggers the protective response against oxidative damage. Overexpression of NRF2 using an adeno-associated virus in the retina of three RP mouse models (Rd1, Rd10 and Rho^−/−^) results in decreased oxidative stress, survival of cone cells and improved visual function [16]. These data suggest antioxidant therapy may help preserve cone function in RP patients.

Gypenosides (Gyp) are major functional saponins in *Gynostemma pentaphyllum*, which has been widely used as a traditional herbal medicine to treat different types of diseases in China and other Asian countries [17]. Gyp have been shown to have anti-oxidative stress, anti-inflammation, anti-apoptotic, anti-diabetic, anti-hyperlipidemic, anti-atherosclerotic and anti-cancer properties in cell lines and in animal models [17]. The protective or toxic effect presented by Gyp is dependent upon the type of disease model and is mediated by different signaling pathways [17]. For example, Gyp demonstrates protective effects against oxidative stress and inflammation at low concentrations, predominantly through the NRF2 and NF-κB pathways [18]. In contrast Gyp also has a toxic effect against cancer at high concentrations by inducing apoptosis and cell cycle arrest and through the inhibition of proliferation and DNA repair [17]. Recent studies have shown Gyp inhibited H_2_O_2_-induced oxidative damage and inflammation in the retinal pigment epithelial (RPE) and the ganglion cells [18,19]. Gyp also reduces inflammation, demyelination and axonal loss in the optic nerve of the optic neuritis mouse model [20]. In a previous study we utilized an *rpgrip1* mutant zebrafish model, which showed early retinal degeneration with only a few rod cells remaining at one-month of age, followed by the death of cone cells [9]. The *rpgrip1* mutant zebrafish mirrors many of the clinical features of RP and the phenotypes of rodent RP models [2,9,10,13,14,15]. In a subsequent paper we found that Gyp inhibited oxidative damage and inflammation and attenuated rod cell death in *rpgrip1* mutant zebrafish [21], however, it remains uncertain as to whether Gyp has a protective effect upon cone cell death. In the current study, we examine whether Gyp protects against cone cell death in the *rpgrip1* mutant zebrafish.

## 2. Materials and Methods

### 2.1. Zebrafish Treatment with Gyp

All zebrafish work were approved by the UK Home Office (Project licence PPL. 70/8697 and ethical protocol code 03/GMA/XS). Zebrafish were maintained in a ZebTec stand-alone system (www.tecniplas.it, accessed on 20 April 2021) at 28 °C. Gyp, containing several gypenoside components, was ordered from Xi’an Jiatian Biotech Co. Ltd., Xi’an, China (98% purity). For oral administration of Gyp, zebrafish were anesthetized by immersion in 0.016% tricaine solution. The anesthetized zebrafish were vertically propped in an E3 medium-soaked sponge. Gyp was dispensed into the zebrafish mouth using a Hamilton syringe with a 22-gauge needle. The dose of Gyp given for each individual zebrafish was based on their body weight. To determine the appropriate dosage for treatment of zebrafish, wildtype and *rpgrip1* mutant zebrafish at two-months post fertilization (mpf) were orally treated with Gyp dissolved in phosphate-buffered saline (PBS) at concentrations of 50, 100, 150 or 200 µg/g body weight once/week for three weeks). The health and wellbeing (such as swimming behavior and food intake) of the fish were monitored daily. At the end of the initial toxicity test, an ideal dose was chosen for further treatment. Three groups of zebrafish at 2 mpf were setup: wildtype zebrafish, rpgrip1 mutant zebrafish and *rpgrip1* mutant zebrafish orally treated with Gyp (50 µg/g body weight, once/week for 16 weeks). Gyp were dissolved in PBS, so the wildtype zebrafish group and the *rpgrip1* control group were orally treated with PBS. After the treatment, zebrafish were sacrificed and samples were collected for histology, immunohistochemistry and biochemical analyses.

### 2.2. Histology and Immunostaining

Zebrafish eyes were fixed in 4% paraformaldehyde (PFA) solution for two hours at room temperature and then washed with PBS twice, followed by dehydration with gradient concentrations of ethanol. After dehydration, the samples were immersed in Histo-clear and then in molten paraffin wax. The paraffin embedded eyes were sectioned with a thickness of 7 µm using a microtome. The sections were dewaxed and hematoxylin and eosin stained. Images were captured using a light microscope. For quantification of photoreceptor layer thickness, five eyes from five individual zebrafish from wildtype, *rpgrip1* mutant and Gyp-treated *rpgrip1* mutant group, were sectioned with two retinal sections of each eye chosen for imaging. The thickness of the photoreceptor layer was measured at the location 0.4 mm from the optic nerve head using ImageJ software.

For immunostaining, PFA fixed zebrafish eyes were immersed in 5% (2 h), 15% (2 h) and 30% (2 h) sucrose, embedded in an optimal cutting temperature compound and cryosectioned at 8 µm thickness. The sections were blocked for 30 min at room temperature with 2% bovine serum albumin (BSA) in PBS and then incubated with Zpr-1 antibody (1:200, www.zfin.org, accessed on 20 April 2021), which specifically targets red and green cone arrestin 3a [22] at 4 °C overnight. The sections were washed three times with PBS (5 min each) and were incubated for 1 h at room temperature with a secondary antibody (1:500, FITC-labelled goat anti-mouse IgG, Sigma). The sections were washed with PBS (5 times for 5 min/each) and mounted with Vectashield medium containing 4′,6-diamidino-2-phenylindole (DAPI) to stain the nuclei. Images were taken under confocal microscopy. Signals for cone cells were quantified using a method described in detail elsewhere [23]. Briefly, 10 retinal sections from 5 eyes (2 sections per eye) of the wildtype zebrafish, *rpgrip1* mutants or Gyp-treated *rpgrip1* mutants were chosen. Fluorescent signals in one region (10 µm × 10 µm, under 400× magnification) in the superior side (0.4 mm from the optic nerve head) of the eye were then quantified with ImageJ software.

### 2.3. ROS Measurement

The zebrafish eyes were collected and lenses were taken out. The eye samples were homogenized in a cold buffer (pH 7.4) containing sucrose (0.32 mM), HEPES (20 mM), MgCl_2_ (1 mM) and phenylmethyl sulfonylfluoride (PMSF, 0.5 mM), and centrifuged for 20 min at 15,000× *g* (4 °C). The supernatants were collected and 20 µL of each sample was transferred into 96-well plates, followed by incubation with 20 μg/mL DCFH-DA (6-Carboxy2′,7′-Dichlorofluorescin diacetate, 8.3 µL/well) for 30 min at 37 °C in the dark. The fluorescent signals were detected using a microplate reader (Fluostar Optima, BMG-labtech) at 485 nm (excitation) and 525 nm (emission).

### 2.4. TUNEL Assay

Cell death in zebrafish retinas was detected using TUNEL assay. The paraffin sections were dewaxed with Histo-clear and rehydrated with decreasing concentrations of ethanol. The sections were permeabilized with proteinase K solution (20 µg/mL) for 10 min, washed with PBS for 5 min, then fixed again with 4% PFA for 5 min, followed by washing with PBS for 5 min. The sections were equilibrated using equilibration buffer (100 µL) at room temperature (RT) for 10 min and labelled with the rTdT reaction mix for an hour in a dark humidified incubator (37 °C). The reaction was stopped with 2 × saline-sodium citrate (SSC) and the sections were co-stained with DAPI. Images were captured using confocal microscopy. To quantify cell death, six sections from six eyes (one section per eye) of the wildtype zebrafish, *rpgrip1* mutants or Gyp-treated *rpgrip1* mutants were used. In each section, TUNEL positive photoreceptors in one region (50 µm × 20 µm, long × wide, under 400× magnification) in the superior side (0.4 mm from the optic nerve head) were counted and are presented as a percentage of total photoreceptor number in the same region.

### 2.5. Gene Expression

Total RNA was extracted from zebrafish eye samples with the TRIzol reagent and cDNA was synthesized using a cDNA synthesis kit (Applied Biosystems, Warrington, UK) following the manufacturer’s guidance. Expression of target genes was measured by quantitative real-time PCR (qRT-PCR) with SYBR Green reagent (Thermo Fisher Scientific, Paisley, UK). The relative expression of candidate genes was calculated using a formula of 2^−ΔΔCT^. The sequences for primers used in qRT-PCR are listed in Table S1.

### 2.6. Biochemical Assays

To measure activities of catalase and superoxide dismutase (SOD) and to quantify levels of glutathione (GSH) and malondialdehyde (MDA) in zebrafish eye samples, OxiSelect Superoxide Dismutase Activity Assay (STA-340), OxiSelect Catalase Activity Assay (STA-341), OxiSelect Total Glutathione (GSSG/GSH) Assay (STA-312) and OxiSelect TBARS (MDA Quantitation) Assay (STA-330) were performed according to the manufacturer’s protocols.

### 2.7. Network Pharmacological Analysis

#### 2.7.1. Potential Targets or Genes Screening

For screening active components in herbs, “*Gynostemmae pentaphylli*” was used as keywords searched against the Traditional Chinese Medicine Systems Pharmacology Database and Analysis Platform (https://tcmspw.com/tcmsp.php, accessed on 20 April 2021) [24]. Active components were collected by name and ADME (absorption, distribution, metabolism and excretion) properties, namely, gypenoside, drug-likeness (DL) ≥0.18 and oral bioavailability (OB) ≥30% [25,26]. The effective targets were fished via on-line tool, Swiss Target Prediction (http://swisstargetprediction.ch/, accessed on 20 April 2021), by using active components as baits [27].

Retinitis pigmentosa (RP)-associated genes were collected from DisGeNET (http://www.disgenet.org/, accessed on 20 April 2021) [28] and Online Mendelian Inheritance in Man (OMIM, https://www.omim.org/, accessed on 20 April 2021) by using “retinitis pigmentosa” as researching keyword.

In view of oxidative stress and inflammation having an important role in the pathogenesis of RP, genes related to oxidative stress or inflammation were gathered from DisGeNET (http://www.disgenet.org/, accessed on 20 April 2021) [28] or GeneCards (The Human Gene Database, https://www.genecards.org/, accessed on 20 April 2021) by using “inflammation”, “inflammation disorder”, “oxidative stress”, “oxidative damage” and “oxidative injury” as researching keywords. All sources used for target screening or disease-related gene prediction are listed in Table S2. All targeting gene identifiers were converted into official gene symbols by using the bitr function from the R package clusterProfiler (v 3.17.0) [29] and only human genes were included.

#### 2.7.2. Interaction Network and Enrichment Analysis

To construct a compound-target-disease network, common targets among active compounds, RP and inflammation or oxidative stress were focused on, and the interactions between them were obtained from the STRING (v 11.0, https://string-db.org/, accessed on 20 April 2021) database. The protein interaction was mapped using the STRING database including high-through lab experiments, automated text mining, previous knowledge of the database and co-expression with a minimum required interaction score of 0.40 (medium confidence). To evaluate the centrality of network nodes, topological analysis was performed by NetworkAnalyzer (a plugin for Cytoscape). This obtains parameters of degree, betweenness, closeness and identifies hub genes as potential key genes for Gyp interfering with RP via anti-inflammatory or anti-oxidative capacity. The visualization of the network was fulfilled by Cytoscape 3.8.0 software (https://cytoscape.org/, accessed on 20 April 2021).

To identify the typical biological functions and pathways of potential targets for Gyp treating RP, both gene ontology (GO) functional enrichment analysis and Kyoto Encyclopedia of Genes and Genomes (KEGG) pathway analysis were performed using the *enrichGO* and *enrichKEGG* functions from R package clusterProfiler (v 3.17.0) [29]. The hypergeometric test was conducted for identifying significant enrichment. Terms or pathways with *p* < 0.05 were considered as statistically significant.

### 2.8. Statistical Analysis

Data were collected from three independent experiments and analyzed using the GraphPad Prism software. Statistical comparisons between individual groups were performed using one-way analysis of variance (ANOVA) followed by the Bonferroni test. Data were presented as mean ± SEM (standard error of the mean). *p* < 0.05 was considered to be significant.

## 3. Results

### 3.1. Initial Toxicity Test of Gyp in Zebrafish

To determine the appropriate dosage of Gyp for this study, 2 mpf wildtype and *rpgrip1* mutant zebrafish were orally treated with Gyp at concentration of 50, 100, 150 or 200 µg/g body weight for three weeks. All zebrafish treated with 150 µg/g body weight died within six days while those treated with 200 µg/g body weight died within four days. All zebrafish treated with 100 µg/g body weight were dead within 2 weeks. Zebrafish treated with 50 µg/g Gyp lived for three weeks without any abnormality. Therefore, Gyp at 50 µg/g bodyweight was chosen for further treatment of rpgrip1 mutants. *Rpgrip1* mutant zebrafish were administrated Gyp (50 µg/g body weight) orally every week for 16 weeks. At the end of the treatment period, histology, immunochemistry, gene expression and biochemical assays were performed.

### 3.2. Cone Cell Death Was Decreased in Gyp-Treated rpgrip1 Mutant Zebrafish

**Figure 1:**
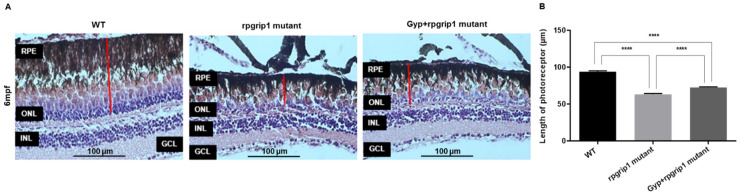
(**A**) Hematoxylin and eosin-stained image of retinal sections of wildtype (WT), *rpgrip1* mutant and Gyp-treated *rpgrip1* mutant zebrafish at 6 mpf. (**B**) Thickness of photoreceptor layer of the three zebrafish groups. Statistical comparisons between individual groups were carried out using one-way ANOVA followed by Bonferroni’s test. Data are displayed as mean ± SEM (*n* = 5 animals of each group). **** *p* < 0.0001. INL, inner nuclear layer; GCL, ganglion cell layer; ONL, outer nuclear layer; RPE, retinal pigment epithelial cells.

*Rpgrip1* mutant zebrafish at 3 mpf demonstrate that almost all rod cells were degenerated, cone cells are morphologically normal though they are not tightly aligned. Degeneration of cone cells was obvious in rpgrip1 mutant zebrafish at 6 mpf [9]. To examine the protective impact of Gyp on cone cell death, *rpgrip1* mutant zebrafish at 2 mpf, before cone cell death starts, were orally treated for 16 weeks till 6 mpf. H&E staining showed that the thickness of the cone photoreceptor layer was significantly reduced by 32.67% in the untreated *rpgrip1* mutant retinas compared to that of wildtype retinas; the thickness of cone photoreceptor layer was significantly increased by 14.74% in *rpgrip1* mutant zebrafish treated with Gyp when compared with that of *rpgrip1* mutants; however, the cone photoreceptor layer in Gyp-treated rpgrip1 mutants was still significantly thinner than that of wildtype zebrafish (Figure 1).

**Figure 2:**
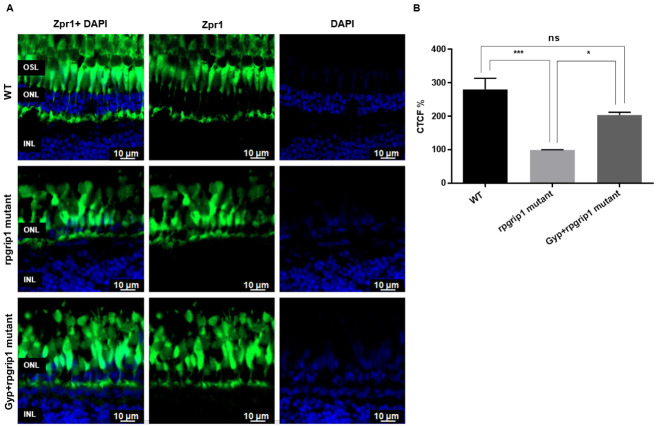
The effect of Gyp treatment on cone degeneration. (**A**) Immunostaining of retinal sections of wildtype, rpgrip1 mutant and Gyp-treated rpgrip1 mutant zebrafish at 6 mpf using zpr-1 antibody where zpr1 stained the cones in green and DAPI labelled nuclei in blue. (**B**) Intensity fluorescence signals (the corrected total cell fluorescence, CTCF) were measured using ImageJ. Statistical comparisons between individual groups were carried out using one-way ANOVA followed by Bonferroni’s test. Data are displayed as mean ± SEM (*n* = 5 animals of each group). Ns, no significance; * *p* < 0.05, *** *p* < 0.001. INL, inner nuclear layer; ONL, outer nuclear layer.

Cone cell degeneration was evaluated by immunostaining zebrafish retinas using the Zpr-1 antibody to detect both red and green cone photoreceptors. The retinas of untreated *rpgrip1* mutant zebrafish demonstrated a significant decrease in the green signal (for both red and green cones) by 64.33% compared to that of wildtype zebrafish retinas; however, in *rpgrip1* mutant zebrafish retinas treated with Gyp, there was a significant increase in the green signal by 29.2% compared to that of untreated *rpgrip1* mutant retinas; green signals in Gyp-treated *rpgrip1* mutant zebrafish eyes was still notably less than that of wildtype zebrafish (Figure 2).

**Figure 3:**
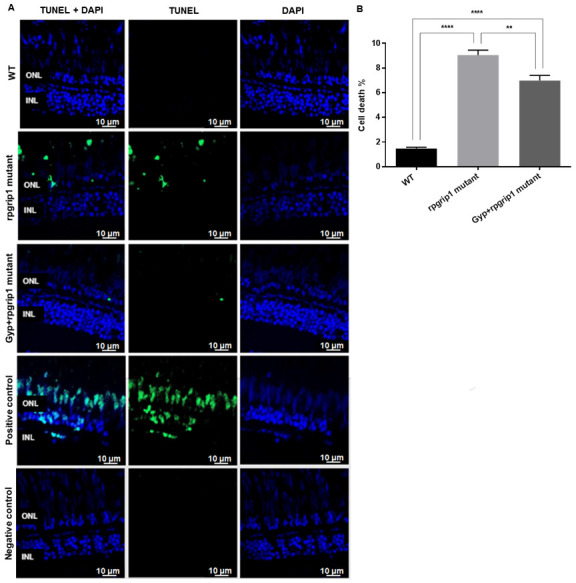
Significant decreases were found in cone apoptosis in rpgrip1 mutant zebrafish treated with Gyp. (**A**) Retinal sections of wildtype (WT), untreated (UT) rpgrip1 mutant and Gyp-treated *rpgrip1* mutant zebrafish at 6 mpf were stained with TUNEL reagents. Nuclei of apoptotic cone cells were stained in green. DAPI labelled nuclei were stained in blue. (**B**) Quantification of apoptotic cells in above retinal sections were compared between the wildtype, *rpgrip1* mutant and Gyp-treated *rpgrip1* mutant zebrafish. Statistical comparisons between individual groups were carried out using one-way ANOVA followed by Bonferroni’s test. Data are displayed as mean ± SEM (*n* = 6 animals of each group). ** *p* < 0.01, **** *p* < 0.0001. INL, inner nuclear layer; ONL, outer nuclear layer.

Death of cone cells was also detected by TUNEL staining, which can detect apoptotic and non-apoptotic cell death. The results demonstrated a significantly increased cell death in the photoreceptor layer of untreated *rpgrip1* mutant zebrafish compared to that of wildtype zebrafish; Gyp treatment led to a significant decrease in cell death compared to that of untreated *rpgrip1* mutants; however, there was significant higher cone cell death in GYP-treated *rpgrip1* mutants than that of wildtype zebrafish (Figure 3). The apoptotic cell death pathways involve caspase-3 and caspase-9 [30,31,32], so we performed qRT-PCR to detect expression of the two caspase genes. Both caspase3 and caspase 9 had markedly increased expression in *rpgrip1* mutant eyes, compared to that of wildtype zebrafish; Gyp treatment resulted in a significant decrease in expression of both genes, compared to that of untreated *rpgrip1* mutants; however, expression of both genes in Gyp-treated *rpgrip1* mutant eyes remained higher than that of wildtype zebrafish (Figure S1).

### 3.3. Gyp Treatment Decreased ROS Production and Increased Antioxidant Capacity in rpgrip1 Mutant Zebrafish

**Figure 4:**
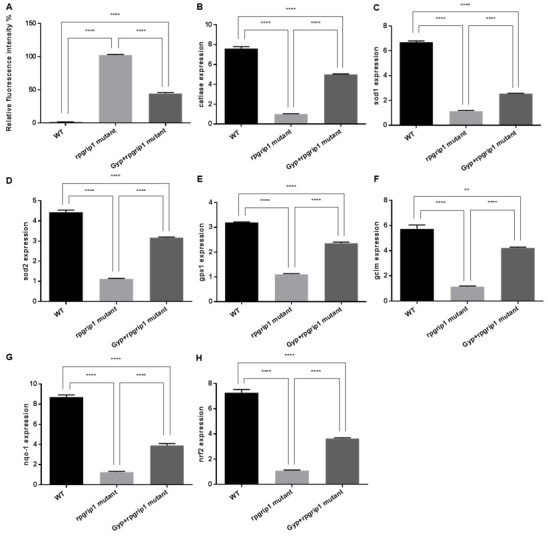
Effects of Gyp on antioxidant capacity in rpgrip1 mutant zebrafish eyes. (**A**) ROS generation in the eyes of wildtype (WT), untreated (UT) and Gyp-treated *rpgrip1* mutant zebrafish at 6 mpf. (**B**–**H**) Expression of *catalase*, *sod1*, *sod2*, *gpx1*, *gclm*, *nqo-1*, *gclm* and *nrf2* was determined using qRT-PCR. Statistical comparisons between individual groups were carried out using one-way ANOVA followed by Bonferroni’s test. Data are displayed as mean ± SEM (*n* = 6 animals of each group). ** *p* < 0.01, **** *p* < 0.0001.

It has been suggested that rod degeneration leads to increased ROS in the outer retina [2]. Similarly, there was a significant increase in ROS production in *rpgrip1* mutant eyes compared to that of wildtype zebrafish; Gyp treatment significantly reduced ROS production compared to that of untreated *rpgrip1* mutants, though ROS level in Gyp-treated eyes higher than that of wildtype zebrafish (Figure 4A).

The effect of Gyp treatment on the expression of the antioxidant genes was assessed by qRT-PCR. In *rpgrip1* mutant zebrafish eyes, catalase expression was significantly reduced compared to that of wildtype zebrafish; in *rpgrip1* mutant zebrafish treated with Gyp, catalase expression was significantly increased compared to that of untreated *rpgrip1* mutants; however, Gyp-treated *rpgrip1* mutant eyes had lower expression of catalase than that of wildtype zebrafish (Figure 4B). Similarly, expression of *sod1*, *sod2*, *gpx1*, *gclm*, *nqo1* and *nrf2* was significantly reduced in *rpgrip1* mutant zebrafish eyes compared to that of wildtype zebrafish; while Gyp treatment significantly increased expression of these genes compared to that of untreated mutant zebrafish, though Gyp-treated mutant zebrafish still had lower expression of the examined genes than that of wildtype zebrafish (Figure 4C–H).

**Figure 5:**
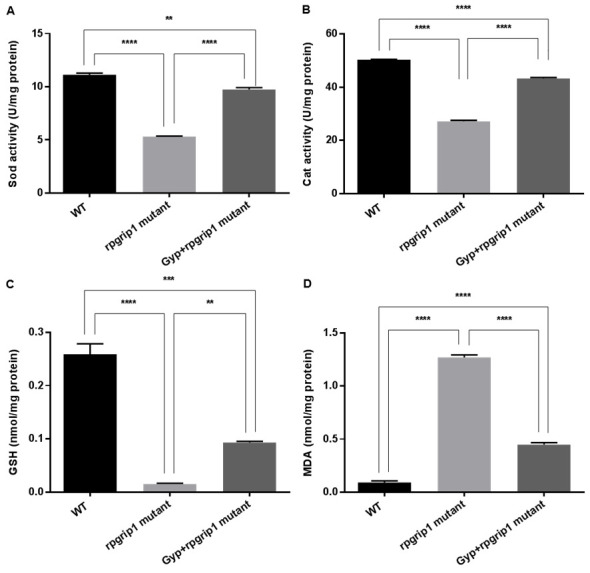
Sod (**A**) and catalase (**B**) activities, and GSH level (**C**) were markedly increased but MDA level (**D**) was significantly decreased in Gyp-treated *rpgrip1* mutant zebrafish eye samples. WT, wildtype zebrafish; UT, untreated rpgrip1 mutant zebrafish. Statistical comparisons between individual groups were carried out using one-way ANOVA followed by Bonferroni’s test. Data are displayed as mean ± SEM (*n* = 6 animals of each group). ** *p* < 0.01, *** *p* < 0.001, **** *p* < 0.0001.

Activities of SOD and catalase and levels of GSH and MDA were also measured in the eyes of wildtype, GYP-treated and untreated *rpgrip1* mutant zebrafish. *Rpgrip1* mutant zebrafish had significantly decreased activities of SOD and catalase compared to that of wildtype zebrafish; GYP treatment resulted in significantly increased activities of SOD and catalase, compared to untreated *rpgrip1* mutants; however, GYP-treated mutants had lower activities of SOD and catalase compared to wildtype zebrafish (Figure 5A,B). In untreated *rpgrip1* mutant zebrafish eyes, GSH level was significantly reduced compared to that of wildtype zebrafish; Gyp-treated zebrafish had significantly increased GSH levels compared to that of untreated mutant zebrafish, though it was significantly lower than that of wildtype zebrafish (Figure 5C). MDA level was significantly increased in *rpgrip1* mutant zebrafish eyes compared to that of wildtype zebrafish; Gyp treatment led to a significant decrease in MDA level compared to that of untreated *rpgrip1* mutant zebrafish; Gyp-treated *rpgrip1* mutants had higher MDA levels than that of wildtype zebrafish (Figure 5D).

### 3.4. Gyp Treatment Suppressed Inflammation in rpgrip1 Mutant Zebrafish Eyes

**Figure 6:**
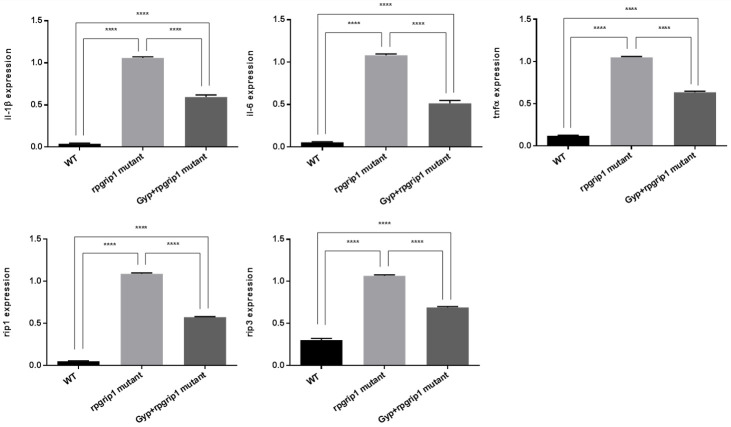
Gyp treatment reduced the expression of inflammation associated genes in *rpgrip1* mutant zebrafish eye samples. Expression of *il-1β*, *il-6*, *tnfα*, *rip1* and *rip3* in eyes of wildtype (WT), untreated (UT) and Gyp-treated *rpgrip1* mutant zebrafish at 6 mpf was determined by qRT-PCR. Statistical comparisons between individual groups were carried out using one-way ANOVA followed by Bonferroni’s test. Data are displayed as mean ± SEM (*n* = 6 animals of each group). **** *p* < 0.0001.

Oxidative stress is linked to inflammation, in that oxidative stress modifies substrates to induce expression of proinflammatory cytokines [18,33]. Expression of inflammatory-related genes in eyes of wildtype, *rpgrip1* mutant and Gyp-treated mutant zebrafish was evaluated using qRT-PCR. In *rpgrip1* mutant zebrafish eyes, expression of *il-1β*, *il-6* and *tnfα* was significantly increased, compared to wildtype zebrafish; Gyp-treated *rpgrip1* mutants had a significant decrease in expression of *il-1β*, *il-6* and *tnfα*, compared to untreated *rpgrip1* mutants; When compared to wildtype zebrafish, Gyp-treated *rpgrip1* mutants had higher expression of the three cytokine genes (Figure 6).

Receptor-interacting serine/threonine protein kinases (RIP) 1 and RIP3 play a critical role in regulating inflammation and cell death [34]. We also found that expression of both *rip1* and *rip3* was markedly increased compared to that of wildtype zebrafish; Gyp-treatment resulted in significantly decreased expression of *rip1* and *rip3* compared to that of untreated *rpgrip1* mutants; however, expression of *rip1* and *rip3* in Gyp-treated rpgrip1 mutants was higher than that of wildtype zebrafish (Figure 6).

### 3.5. Gyp Attenuated Expression of Endoplasmic Reticulum (ER) Stress Associated Genes in rpgrip1 Mutant Eyes

ROS production and ER stress are two interactive events, associated with a wide range of human diseases [35,36]. ER stress activates the unfolded protein response (UPR), containing PERK, IRE1 and ATF6 signaling pathways. We examined expression of the UPR components including *bip*, *atf4*, *atf6* and *xbp1* in eyes of wildtype, *rpgrip1* mutant and Gyp-treated mutant zebrafish. Expression of *bip*, *atf4*, *atf6*, *xbp1t* (total) and *xbp1s* (spliced variant) was markedly increased in *rpgrip1* mutant eyes compared to that of wildtype zebrafish; Gyp-treatment led to significantly decreased expression of these genes compared to that of untreated rpgrip1 mutants; expression of *bip* and *xbp1s* (but not *atf4*, *atf6* or *xbp1t*) remained higher in Gyp-treated mutants compared to that of wildtype zebrafish (Figure S2).

### 3.6. Network Pharmacological Analysis

To identify the potential active compounds in herbs, firstly, “*Gynostemmae Pentaphylli*” was searched against the TCMSP database. Based on the ADME parameters of DL ≥ 0.18 and OB ≥ 30%, a total of 24 active compounds were identified. Among these, 9 gypenosides were included. The main properties of Gyp are listed in Table S3. Subsequently, potential targets were predicted using Swiss Target prediction, and the top 15 targets of each gypenoside were kept and merged. After unifying the gene symbols and dereplication, a total of 63 targets of gypenosides were finally screened out (Figure S3). The RP-related genes were gathered from DisGeNET and OMIM. After normalizing the gene symbols and removing duplicates, a total of 745 RP-related genes were finally identified. Furthermore, 1132 and 745 unique genes related to oxidative stress and inflammation were collected from GeneCards and DisGeNET database, respectively.

**Figure 7:**
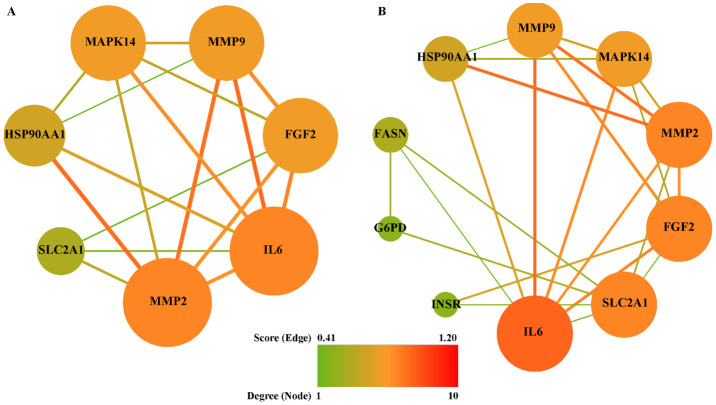
PPI network between common targets. (**A**) Inflammation, (**B**) oxidative stress. The nodes represented targets and edges represented interaction among targets. The node size and color were represented with degree, while edge size and color were represented with combined score.

To explore the link between Gyp and RP in oxidative stress and inflammatory aspects, 7 and 11 common targets among potential targets of gypenosides, RP-related genes, inflammation-related genes or oxidative stress-related genes were extracted, respectively. Subsequently, two PPI networks (Figure 7) were constructed by employing online tool STRING. Among the common targets, SIGMAR1 was not displayed in Figure 7, because it was not connected with the network. The shared targets among gypenosides, RP and inflammation were MMP2, IL-6, FGF2, MMP9, MAPK14, HSP90AA1 and SLC2A1, are shown in Figure 7A. The connection of Gyp, RP and oxidative stress is illustrated in Figure 7B. Among these mapped interactions, MMP2-HSP90AA1 and MAPK14-HSP90AA1 were taken from a verified experimental source (Figure 7A). Similarly, FGF2-INSR and SLC2A1-INSR belong to verifiable experimental interactions (Figure 7B). According to topological network analysis (Tables S4 and S5), both MMP2 and IL-6 had the highest degree, and were identified as hub genes. In addition, FGF2, SLC2A1, MMP9 and MAPK14 were also suggested to be crucial nodes in the networks. Owing to the common targets shared by gypenosides, RP and inflammation being fully included in that shared by gypenosides, RP and oxidative stress, all common targets were combined into one set.

To investigate the multiple functions of the common targets (Figure 7), GO molecular function enrichment analysis was conducted. The top 6 significantly enriched GO terms (*p* < 0.05) were predominantly involved in protein binding (GO:0005515), identical protein binding (GO:0042802), metallopeptidase activity (GO:0008237) and protein tyrosine kinase activity (GO:0004713) (Table S6). In addition to GO enrichment, the KEGG pathway was also analyzed to clarify the potential mechanism of gypenosides treating RP via anti-oxidative stress or anti-inflammation. The candidate common targets were principally enriched in pathways in cancer (hsa05200 and hsa05205), the HIF-1 signaling pathway (hsa04066), the TNF signaling pathway (hsa04668), the PI3K-Akt signaling pathway (hsa04151) and leukocyte transendothelial migration (hsa04670) (Table S7). In addition, the hub genes identified from GO enrichment analysis are similar to those in KEGG signalling pathways, including FGF2, IL-6, MAPK14, MMP2, MMP9 and SLC2A1 (Tables S6 and S7).

## 4. Discussion

The retinal pathology of *rpgrip1* mutant zebrafish resembles that of RP patients who show initial rod cell death and subsequent cone cell death. So *rpgrip1* mutant zebrafish is an excellent platform for the development of new treatments for RP. Here we have demonstrated that Gyp administration reduces cone cell death, enhances antioxidant capacity, suppresses inflammation and inhibits ER stress in *rpgrip1* mutant zebrafish. Network pharmacological analysis further suggests that the protective effects of Gyp are mediated by inhibition of oxidative stress and inflammation.

Early studies demonstrate that exposure to high oxygen levels for several weeks results in mouse photoreceptor degeneration. Increased photoreceptor death was found in the central retinal region, where the highest amount of oxygen is supplied from the choroidal blood flow [37]. In RP, the progressive death of rod cells leads to decreased oxygen utilization and a resultant increase in oxygen tension in the outer retina. In the Royal College of Surgeons (RCS) rats, rod photoreceptors are completely degenerated by postnatal (P) 30. Oxygen tension in the outer retina was increased from P25 and after P30 the cones endured oxygen tension as high as four times compared to that of the normal rats [38]. Similarly, significantly higher oxygen tension has been reported in the outer retinas of P23H rats and of an RP cat model [39,40]. Exposure to higher levels of oxygen tension is likely to cause oxidative damage to cone cells, resulting in the gradual death of cone cells. Acrolein and 4-hydroxynonenal, the biomarkers for oxidative damage, have been detected at higher levels in the cone cells of the RHODOPSINPro347Leu transgenic pigs at 10 months old, when all the rod cells have completely degenerated [12]. Acrolein has also been detected in the cone cells of rd1 mice at P35, about two weeks after degeneration of most rod cells [13]. Here we also found a significantly higher level of ROS in *rpgrip1* mutant eyes at 6 mpf (Figure 4A), when only cones were survived and degeneration of cone cells was evident [9]. The antioxidant defense system was also compromised in *rpgrip1* mutant eyes, demonstrating a marked decrease in expression of antioxidant genes and in activities of antioxidant enzymes (Figure 4 and Figure 5). Previous studies have shown Gyp’s protection against oxidative damage in vitro and in vivo [18,19,21,41,42,43,44]. Here we have demonstrated that Gyp treatment can suppress ROS production, upregulated antioxidant gene expression and increased antioxidant enzyme activities and GSH level in *rigrip1* mutant eyes (Figure 4 and Figure 5). Similar to the other antioxidants which protect against cone death in RP models [12,13,14,15], Gyp treatment also attenuated cone death in *rpgrip1* mutant zebrafish (Figure 2 and Figure 3).

It is well known that oxidative stress triggers inflammation in chronic diseases such as RP [45]. RP patients have shown significantly high levels of proinflammatory cytokines and chemokines in the vitreous cavity and aqueous humor [46]; previous studies also reported increased expression of proinflammatory cytokines (e.g., IL-1β and TNFα) and microglial activation in the retinas of RP mouse models [23,47]. In rd1 mice at P40, when only cone cells are survived, the retinas had significantly high expression of *Il-1α*. *Il-1β*, *Tnfα*, *C1qα* and *Tmem119* (a marker for microglial activation); however, rd1 mice treated with PLX5622, which eliminates microglia, showed similar levels of expression of those genes in the retinas as that of wildtype mice, indicating activated microglia mediated expression of these genes [48]. We also detected higher expression of proinflammatory genes (*il-1β*, *il-6* and *tnfα*) in *rpgrip1* mutant eyes at 6 mpf, when only cone cells are survived. Gyp treatment significantly reduced expression of *il-1β*, *il-6* and *tnfα*. NF-κB pathway plays an important role retinal inflammation and is involved in RP-associated cone cell death [49]. We, and others, have shown that Gyp suppresses inflammation via inhibiting activation of the NF-κB pathway [50,51]. So downregulation of proinflammatory gene expression by Gyp in *rpgrip1* mutant eyes is possibly mediated by inactivation of NF-κB pathway.

We further utilized network pharmacology to clarify the possible mechanisms of gypenosides against photoreceptor death in RP. Based on the predicted gene associations from the PPI network, IL-6, MMP2, MMP9, MAPK14, SLC2A1 and FGF2 were identified as main hub genes, which are potentially involved in the treatment of RP using Gyp via an anti-oxidative stress or anti-inflammation route. IL-6 plays important roles in inflammation, infection responses, immunity and disease [52]. Increased IL-6 expression has been detected in intraocular fluid of RP patients and in the retinas of RP animal models [46,53,54]. We also detected a high level of il-6 expression in *rpgrip1* mutant eye, suggesting IL-6 contributes to the pathogenesis of RP. IL-6 can enhance the generation of matrix metalloproteinases (MMPs) [55]. MMP-2 and MMP-9 were members of gelatinase family and zinc-dependent enzymes, with capability of decomposing the extracellular matrix and basement membrane components. MMP2 has been detected at significantly high levels in the aqueous humor of RP patients [56] and inhibition of MMP9 has been found to suppress rod cell death in a rat RP model [57]. However, the underlying MMP2 and MMP9 associated mechanisms with RP are not clear and need further attention. Mitogen-activated protein kinases (MAPKs) belonged to the family of evolutionarily conserved serine/threonine kinases, which mediated the regulation of multiple cell processes, including cell growth, differentiation, autophagy and apoptosis. The MAPK signaling pathway has been involved in RP pathogenesis. A recent study demonstrated that inhibition of histone deacetylases attenuated secondary cone death via regulation of MAPK and PI3K-Akt signaling pathways [58]. Solute carrier family 2 member 1 (SLC2A1), also known as glucose transporter 1 (GLUT1), is closely associated with cellular energy metabolism. The protection of rod-derived cone viability factor (RdCVF) against cone death in RP mouse models is mediated by promotion of GLUT1 transport of glucose into cone cells and stimulation of aerobic glycolysis [59]. FGF2, as a pleiotropic heparin-binding factor, has angiogenic, mitogenic and immunomodulatory activities. Early studies have demonstrated that FGF2 delays photoreceptor degeneration in RP rat models [60,61,62,63]. Recently, Di Pierdomenico reported that FGF2 suppressed cone cell death in RP rats [64].

The GO functional analysis uncovered the associated functions of common targets. These GO terms gathered core hub targets (Table S6) which were shared by Gyp, RP, oxidative stress and inflammation. The pathway enrichment analysis demonstrated that the HIF, TNF and PI3K-AKT signaling pathways were the most enriched and related to RP and Gyp-mediated protection (Table S7). The TNF signaling pathway is particularly critical in photoreceptor death. TNFα mediates photoreceptor apoptosis, necroptosis and pyroptosis in RP [65]. It is also reported that TNFα knockdown in RP mouse models promotes cone survival [66]. The data from network pharmacology analysis suggest that the protection of Gyp against photoreceptor death is mediated by the main hub genes-associated signaling pathways. However, the underlying mechanisms remain elusive.

Our data were mainly gathered from molecular biology, biochemistry and histology approaches. Ideally, we would combine retinal biochemical/histological changes with retinal visual functions. The data from network pharmacology analysis will need to be validated in RP animal models. Additionally, the protection of Gyp against rod and cone degeneration requires further evaluated in other RP models. Data from preclinical studies will guide future clinic trials of Gyp-treating RP patients.

## 5. Conclusions

Gyp decreased ROS production, increased antioxidant capacity and suppressed ER stress and inflammation; resulting in increased cone survival in *rpgrip1* mutant zebrafish. The delay of cone degeneration by Gyp is possibly mediated by multiple signaling pathways. Gyp has therapeutic potential for RP patients.

## Data Availability

Data is contained within the article or supplementary material.

## Supplementary Materials

The following are available online at https://www.mdpi.com/article/10.3390/antiox10071050/s1, Figure S1: Expression of *caspase-3* and *caspase-9* genes in eyes of wildtype (UT), untreated (UT) and Gyp-treated rpgrip1 mutant zebrafish; Figure S2: Expression of ER stress related genes: *bip*, *atf4*, *atf6, xbp1t* and *xbp1s* in eyes of wildtype (WT), untreated and Gyp-treated rpgrip1 mutant zebrafish; Figure S3: Interaction network between 9 gypenosides and 63 their potential targets. Table S1: Primers used for qRT-PCR; Table S2: Sources used for targets or genes screening; Table S3: Main active components of *Gynostemmae pentaphylli*; Table S4: Topological network analysis of common targets among gypenosides, inflammation and retinitis pigmentosa;Table S5: Topological network analysis of common targets among gypenosides, oxidative stress and retinitis pigmentosa; Table S6: Gene ontology (GO) enrichment analysis of common targets among gypenosides, inflammation, oxidative stress and retinitis pigmentosa; Table S7: Kyoto Encyclopedia of Genes and Genomes (KEGG) enrichment analysis of common targets among gypenosides, inflammation, oxidative stress and retinitis pigmentosa.
