# Supplementary material for: Gypenosides Alleviate Cone Cell Death in a Zebrafish Model of Retinitis Pigmentosa
Source: Antioxidants (Basel). 2021 Jun 29;10(7):1050. doi: 10.3390/antiox10071050 (PMC8300748; doi:10.3390/antiox10071050)
Supplement: Supplementary file 1 [file antioxidants-10-01050-s001.zip › antioxidants-1232584-supplementary.pdf]

Supplementary data

Figure S1

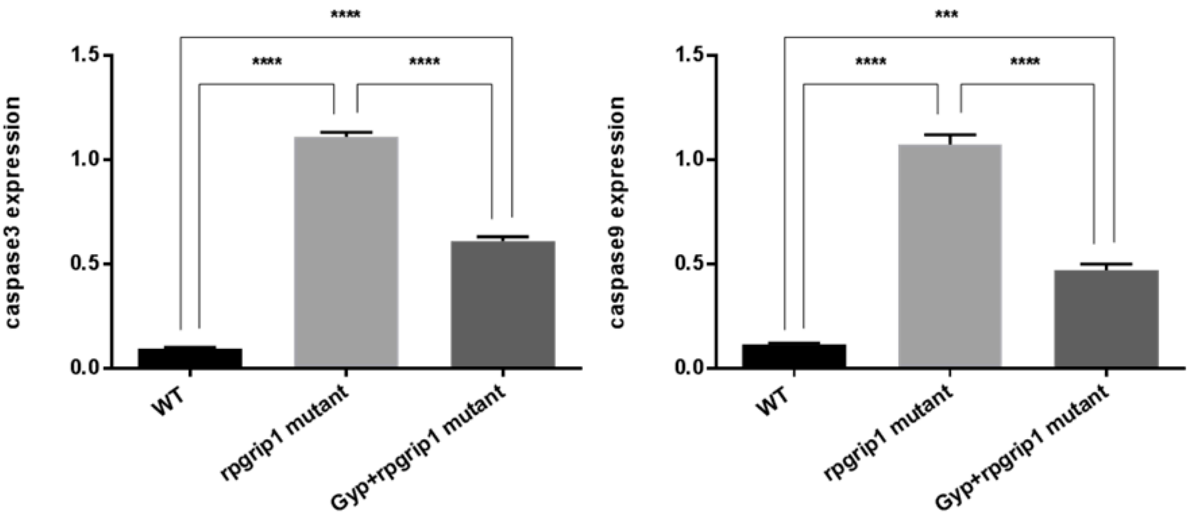

Figure S1 Expression of *caspase-3* and *caspase-9* genes in eyes of wildtype (UT), untreated (UT) and Gyp-treated rpgrip1 mutant zebrafish at 6mpf, detected by qRT-PCR. ***p<0.001, ****p<0.0001.

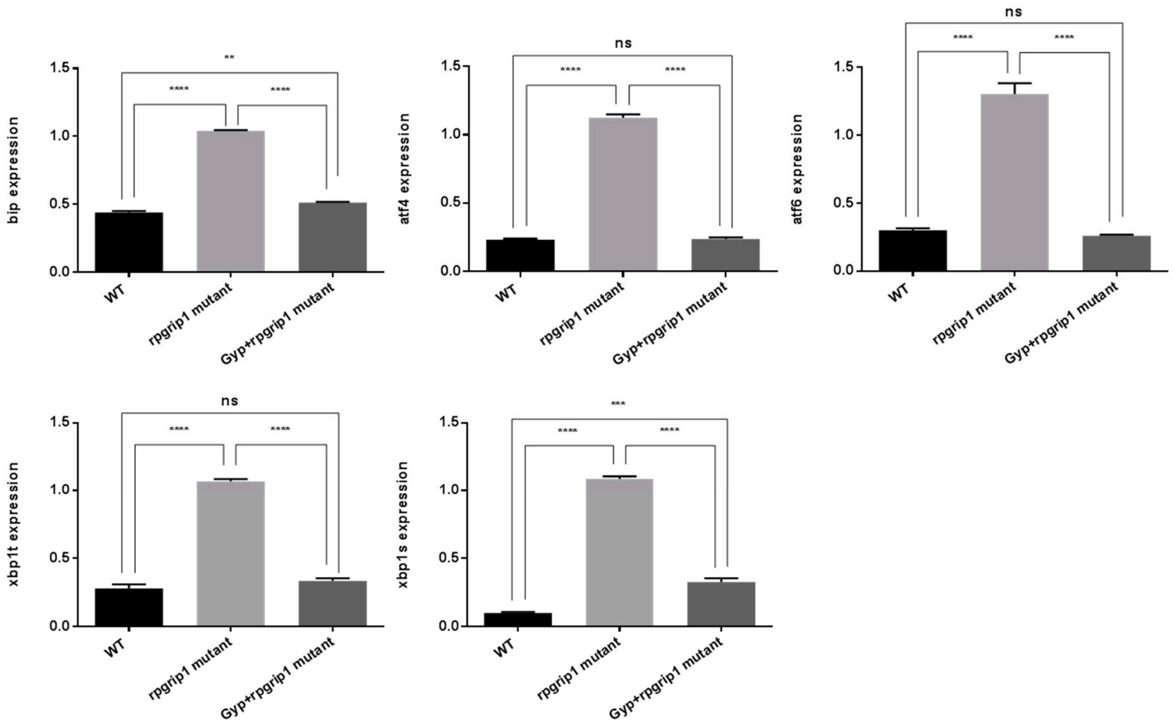

Figure S2 Expression of ER stress related genes: *bip*, *atf4*, *atf6, xbp1t* and *xbp1s* in eyes of wildtype (WT), untreated and Gyp-treated rpgrip1 mutant zebrafish at 6mpf, determined by qRT-PCR. Ns, no significance; **p<0.01, ***p<0.001, ****p<0.0001.

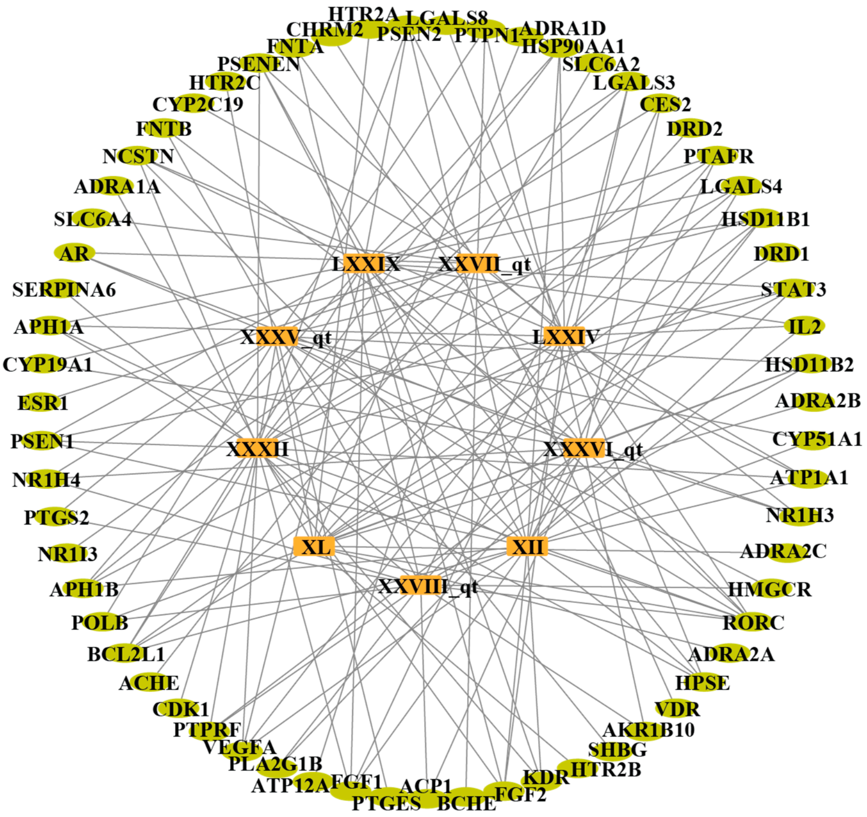

Figure S3 Interaction network between 9 gypenosides and 63 their potential targets. Dark green nodes stand for gene symbols of targets. Orange nodes represent individual gypenosides.

Table S1 Primers used for qRT-PCR

Gene	Primer sequence 5' - 3'	TM	Band size (bp)	Reference
β-actin	F=ACTGTATTGTCTGGTGGTAC<br>R=TACTCCTGCTTGCTAATCC	60°C	197	Soares et al., 2012
Sod1	F=CGCATGTTCCCAGACATCTA<br>R=GAGCGGAAGATTGAGGATTG	53.9°C	100	Timme-Laragy et al., 2009
Sod2	F= CTAGCCCGCTGACATTACATC<br>R= TTGCCCACATAGAAATGCAC	54.5°C	101	Timme-Laragy et al., 2009
catalase	F=TGAGGCTGGGTCATCAGATA<br>R=AAAGACGGAAACAGAAGCGT	54.95°C	138	Zhu and Chan., 2012
nrf2	F=GAGCGGGAGAAATCACACAGAATG<br>R= CAGGAGCTGCATGCACTCATCG	59.25°C	85	Timme-Laragy et al., 2012
gpx1	F=AGGCACAACAGTCAGGGATT<br>R=CAGGAACGCAAACAGAGGG	56.45°C	241	Hou et al., 2015
nqo-1	F=AACGCCGCTGCGCGAGATGTTG<br>R=TCCATTCCTCCAGTGGTGAAGG	62°C	446	Kobayashi et al., 2002
gclm	F=TCCAGATCTCACTGCATTCG<br>R=ATGCCTCTGCTCTTGACGAT	55.4°C	186	Designed in lab
tnf-α	F= ACCAGGCCTTTTCTTCAGGT<br>R= GCATGGCTCATAAGCACTTGTT	56.6°C	148	Designed in lab
il-6	F= TCAACTTCTCCAGCGTGATG<br>R= TCTTTCCCTCTTTTCCTCCTG	54.9°C	73	Designed in lab
il-1β	F= TTCCCCAAGTGCTGCTTATT<br>R= AAGTTAAAACCGCTGTGGTCA	54.6°C	149	Designed in lab
xbp1s	F= TGTTGCGAGACAAGACGA<br>R= CCTGCACCTGCTGCGGACT	60°C	185	Vacaru., et al. 2014
xbp1t	F= GAGGAGCCCACAAAGTCCTC<br>R= CGAAGTGCTTTTTCCTCTGG	60°C	196	Vacaru., et al. 2014
atf4	F= TTAGCGATTGCTCCGATAG<br>R= GCTGCGGTTTTATTCTGCTC	54.60°C	208	Vacaru., et al. 2014

atf6	F= CTGTGGTGAAACCTCCACC<br>R= CATGGTGACCACAGGAGATG	57.98°C	200	Vacaru., et al. 2014
bip	F= AAGAGGCCGAAGAGAAGGAC<br>R= AGCAGCAGAGCCTCGAAATA	59.9°C	133	Vacaru., et al. 2014
Caspase3	F= TAGTGTGTGTGTTGCTCAGTC<br>R= CTCGACAAGCCTGAATAAAG	56.16°C	153	Chen et al., 2014
Caspase9	F= CTGAGGCAAGCCATAATCG<br>R= AGAGGACATGGGAATAGCGT	57.19°C	248	Zhang et al., 2016
rip1	F= GATCTCTCGGCTTGTAGCATGA<br>R= ATATGTACAGAGCAGGGAGAAAACTAACA			Wang et al., 2011
rip3				

F: forward; R: reverse

Table S2 Sources used for targets or genes screening.

	Keywords / Parameters	Database/Tool	Filtering
**Active components**	*Gynostemmae Pentaphylli*	TCMSP (https://tcmspw.com/tcmsp.php)	Gypenoside, DL⩾0.18, OB ⩾30%
	Structure file (.mol2 format)	Swiss Target Prediction (http://swisstargetprediction.ch/)	Keep top 15
**Disease**	Retinitis pigmentosa	DisGeNET (http://www.disgenet.org/)<br>OMIM (https://www.omim.org/)	
**Inflammation**	Inflammation; Inflammatory disorder	DisGeNET (http://www.disgenet.org/)	
**Oxidative stress**	Oxidative stress; Oxidative injury; Oxidative damage	GeneCards (https://www.genecards.org/)	

Table S3 Main active components of *Gynostemmae Pentaphylli*.

Molecule ID	Molecule name	Molecular weight	OB(%)	DL
MOL009888	Gypenoside XXXVI_qt	458.80	37.85	0.78
MOL009928	Gypenoside LXXIV	801.14	34.21	0.24
MOL009929	Gypenoside LXXIX	785.14	37.75	0.25
MOL009938	Gypenoside XII	785.14	36.43	0.25
MOL009943	Gypenoside XL	799.12	30.89	0.21
MOL009969	Gypenoside XXXV_qt	444.77	37.73	0.78
MOL009971	Gypenoside XXVII_qt	418.73	30.21	0.74
MOL009973	Gypenoside XXVIII_qt	416.71	32.08	0.74
MOL009976	Gypenoside XXXII	787.11	34.24	0.25

Table S4 Topological network analysis of common targets among gypenosides, inflammation and retinitis pigmentosa

Gene symbol	Gene name	Degree	Closeness	Betweenness
MMP2	Matrix metalloproteinase-2	6	1.000	0.094
IL6	Interleukin-6	6	1.000	0.094
FGF2	Fibroblast growth factor 2	5	0.857	0.044
MAPK14	Mitogen-activated protein kinase 14	5	0.857	0.017
MMP9	Matrix metalloproteinase-9	5	0.857	0.017
HSP90AA1	Heat shock protein HSP 90-alpha	4	0.750	0.000
SLC2A1	Solute carrier family 2	3	0.667	0.000

Table S5 Topological network analysis of common targets among gypenosides, oxidative stress and retinitis pigmentosa

Gene symbol	Gene name	Degree	Closeness	Betweenness
IL6	Interleukin-6	7	0.818	0.206
SLC2A1	Solute carrier family 2	6	0.750	0.250
MMP2	Matrix metalloproteinase-2	6	0.750	0.071
FGF2	Fibroblast growth factor 2	6	0.750	0.134
MMP9	Matrix metalloproteinase-9	5	0.643	0.012
MAPK14	Mitogen-activated protein kinase 14	5	0.643	0.012
HSP90AA1	Heat shock protein HSP 90-alpha	4	0.562	0.000
FASN	Fatty acid synthase	3	0.600	0.037
G6PD	Glucose-6-phosphate 1-dehydrogenase			
INSR	Insulin receptor			
SIGMAR1	Sigma non-opioid intracellular receptor 1			

Table S6 Gene ontology (GO) enrichment analysis of common targets among gypenosides, inflammation, oxidative stress and retinitis pigmentosa

Term	P-Value	Enrichment Gene
GO:0004713~ protein tyrosine kinase activity	0.0027	FGF2, HSP90AA1, INSR
GO:0042802~ identical protein binding	0.0083	SLC2A1, MMP9, HSP90AA1, G6PD
GO:0005515~ protein binding	0.0149	IL6, SLC2A1, MMP2, FGF2, MMP9, HSP90AA1, FASN, G6PD
GO:0019903~ protein phosphatase binding	0.0367	MAPK14, HSP90AA1
GO:0008144~ drug binding	0.0441	FASN, SIGMAR
GO:0008237~ metallopeptidase activity	0.0470	MMP2, MMP9

The hypergeometric test was used for identifying significantly enrichment with $P<0.05$. Core hub targets were highlighted in red.

Table S7 Kyoto Encyclopedia of Genes and Genomes (KEGG) enrichment analysis of common targets among gypenosides, inflammation, oxidative stress and retinitis pigmentosa

Term	P-Value	Enrichment Gene
hsa05200: Pathways in cancer	6.18E-05	IL6, MMP2, FGF2, SLC2A1, MMP9, HSP90AA1
hsa05205: Proteoglycans in cancer	1.79E-03	MMP2, FGF2, MMP9, MAPK14
hsa04621: NOD-like receptor signaling pathway	2.26E-03	IL6, MAPK14, HSP90AA1
hsa04066: HIF-1 signaling pathway	6.51E-03	IL6, SLC2A1, ISNR
hsa04915: Estrogen signaling pathway	6.91E-03	MMP2, MMP9, HSP90AA1
hsa04668: TNF signaling pathway	8.04E-03	IL6, MMP9, MAPK14
hsa04931: Insulin resistance	8.18E-03	IL6, SLC2A1, ISNR
hsa04151: PI3K-Akt signaling pathway	8.38E-03	IL6, FGF2, HSP90AA1, ISNR
hsa04670: Leukocyte transendothelial migration	9.24E-03	MMP2, MMP9, MAPK14

The top 10 enriched pathways were displayed. The hypergeometric test was used for identifying significantly enrichment with $P<0.05$. Core hub targets were highlighted

References

Chen, T., Wang, H., Zhang, Z., Li, Q., Yan, K., Tao, Q., Ye, Q., Xiong, S., Wang, Y. and Zhai, Z., 2014. A novel cellular senescence gene, SENEX, is involved in peripheral regulatory T cells accumulation in aged urinary bladder cancer. PloS one, 9(2), e87774.

Hou, J., Li, L., Xue, T., Long, M., Su, Y. and Wu, N., 2015. Hepatic positive and negative antioxidant responses in zebrafish after intraperitoneal administration of toxic microcystin-LR. Chemosphere, 120, 729-736.

Kobayashi, M., Itoh, K., Suzuki, T., Osanai, H., Nishikawa, K., Katoh, Y., Takagi, Y. and Yamamoto, M., 2002. Identification of the interactive interface and phylogenic conservation of the Nrf2-Keap1 system. Genes to Cells, 7(8), 807- 820.

Soares, J., Castro, L.F.C., Reis-Henriques, M.A., Monteiro, N.M. and Santos, M.M., 2012. Zebrafish (Danio rerio) life-cycle exposure to chronic low doses of ethinylestradiol modulates p53 gene transcription within the gonads, but not NER pathways. Ecotoxicology, 21(5), 1513-1522.

Timme-Laragy, A.R., Karchner, S.I., Franks, D.G., Jenny, M.J., Harbeitner, R.C., Goldstone, J.V., McArthur, A.G. and Hahn, M.E., 2012. Nrf2b, novel zebrafish paralog of oxidant-responsive transcription factor NF-E2-related factor 2 (NRF2). Journal of Biological Chemistry, 287(7), 4609-4627.

Vacaru, A.M., Di Narzo, A.F., Howarth, D.L., Tsedensodnom, O., Imrie, D., Cinaroglu, A., Amin, S., Hao, K. and Sadler, K.C., 2014. Molecularly defined unfolded protein response subclasses have distinct correlations with fatty liver disease in zebrafish. *Disease models & mechanisms*, *7*(7), 823-835.

Wang, W.L., Hong, J.R., Lin, G.H., Liu, W., Gong, H.Y., Lu, M.W., Lin, C.C. and Wu, J.L., 2011. Stage-specific expression of TNFα regulates Bad/Bid-mediated apoptosis and RIP1/ROS-mediated secondary necrosis in birnavirus-infected fish cells. *PloS one*, *6*(2), p.e16740.

Zhang, Y., Liu, K., Hassan, H.M., Guo, H., Ding, P., Han, L., He, Q., Chen, W., Hsiao, C.D., Zhang, L. and Jiang, Z., 2016. Liver Fatty Acid Binding Protein Deficiency Provokes Oxidative Stress, Inflammation, and Apoptosis-Mediated Hepatotoxicity Induced by Pyrazinamide in Zebrafish Larvae. *Antimicrobial Agents and Chemotherapy*, 60(12), 7347-7356.

Zhu, J.Y. and Chan, K.M., 2012. Mechanism of cadmium-induced cytotoxicity on the ZFL zebrafish liver cell line. Metallomics, 4(10), 1064-1076.
